# Parental Socioeconomic Status and Weight Faltering in Infants in Japan

**DOI:** 10.3389/fped.2018.00127

**Published:** 2018-05-01

**Authors:** Yuko Kachi, Takeo Fujiwara, Yui Yamaoka, Tsuguhiko Kato

**Affiliations:** ^1^Department of Hygiene and Public Health, Nippon Medical School, Tokyo, Japan; ^2^Department of Social Medicine, National Center for Child Health and Development, Tokyo, Japan; ^3^Department of Public Health, Kitasato University School of Medicine, Kanagawa, Japan; ^4^Department of Global Health Promotion, Tokyo Medical and Dental University, Tokyo, Japan; ^5^Center on Child Abuse and Neglect, University of Oklahoma Health Science Center, Oklahoma City, OK, United States

**Keywords:** socioeconomic status, income, education, failure to thrive, weight faltering, birth cohort, infant, Japan

## Abstract

**Background:** Previous studies in the UK and Denmark found no significant association between low socioeconomic status (SES) and weight faltering. However, to our knowledge, there are no studies from other developed countries. We examined the association between parental SES and weight faltering in infants up to 1.5 years of age, and investigated whether the inequalities changed between 2001 and 2010 in Japan.

**Methods:** We used data from two Japanese population-based birth cohorts started in 2001 (*n* = 34,594) and 2010 (*n* = 21,189). Parental SES was assessed as household income and parental education when the infant was 6 months old. Weight faltering was defined as the slowest weight gaining in 5% of all children in each cohort. Logistic regression analyses were conducted with adjustment for covariates. The relative index of inequality was used to assess relative impact of parental SES on weight faltering.

**Results:** Infants in the lowest quartile of household income were 1.29 (95% confidence interval [CI]: 1.10, 1.52) and 1.27 (95% CI: 1.03, 1.56) times more likely to experience weight faltering than those in the highest income quartile both in the 2001 and 2010 cohorts, respectively. The relative index of inequality for household income was 1.66 (95% CI: 1.36, 1.96) in 2001 and 1.86 (95% CI: 1.42, 2.31) in 2010.

**Conclusions:** Infants from lower income families have a greater risk of weight faltering in Japan. Additionally, the income-related inequalities in weight faltering did not change between the two cohorts. Social policies to address maldistribution of weight faltering due to household income are needed.

## Introduction

Weight faltering, or failure to thrive, describes infants and young children who do not gain weight as expected (1). In clinical practice, it is defined as a weight for age below the 5th percentile on multiple occasions or a weight deceleration crossing two major percentile lines on a growth chart (2). In epidemiological research, weight faltering is defined as being among the slowest gaining 5% on a conditional weight gaining chart, which compares an infant's current weight to the predicted weight, based on their previous weight (3). Weight faltering in infancy is associated with subsequent growth delay and cognitive deficiencies (4, 5). Thus, it is essential to identify factors that could aid in the early detection and prevention of weight faltering. Although the causes of weight faltering were traditionally subdivided into organic and nonorganic, it is now recognized that the causes are typically multifactorial (biological, psychosocial, and environmental) (6, 7).

Weight faltering has been seen as a manifestation of poverty (1). While this is still likely to be true in developing countries, previous studies in the UK (8–12) and Denmark (13, 14) consistently reported no significant association between low parental socioeconomic status (SES) and weight faltering. For example, large UK cohort studies (9, 10) suggested that short parental height, high parity, prolonged breast feeding, and feeding problems predicted weight faltering rather than low parental SES. Another UK case-control study (12) suggested that early feeding problems such as slow feeding and weak sucking were the main risk factors of weight faltering, and not parental SES. Two Danish cohort studies (13, 14) indicated no association between area-level SES and weight faltering.

The absence of an association between parental SES and weight faltering observed in these studies probably reflects a social security safety net that assists families with young children (1). Parental SES might be associated with infant weight faltering in other developed countries, including Japan, which do not have adequate social welfare policies for infants. In Japan, social changes such as urbanization and the trend toward nuclear families have weakened informal child-rearing support from the family and community (15). Moreover, many families face economic difficulties due to decreases in income and job security under the stagnant economy (16) while facing the heavy economic burden of child rearing. In Japan, the proportions of family-related social expenditures of the gross domestic product were 0.60% in 2001 and 1.28% in 2010. Despite the slight increase, this is lower than the 2010 budgets of the UK (4.01%) and Sweden (3.63%) (17). SES inequalities in weight faltering may have persisted in Japan as the social security net for families with children has not significantly improved during the past decade.

Therefore, we examined if weight faltering in infants up to 1.5 years of age was more prevalent in socioeconomically disadvantaged families and if these inequalities changed between 2001 and 2010 in Japan.

## Materials and methods

### Data source and study sample

We used data from the first and second surveys of the “Longitudinal Survey of Newborns in the Twenty First Century,” a national birth cohort study of two cohorts (infants born in 2001 and 2010) conducted by the Ministry of Health, Labor and Welfare in Japan in 2001 and 2010 (18). All infants born in Japan between January 10–17 and between July 10–17, 2001 for the first cohort (*n* = 53,575) and between May 10–24, 2010 for the second cohort (*n* = 43,767) were eligible. Infants were identified using Japanese vital statistics birth records.

The first and second questionnaires of the surveys were mailed to parents when the children were 6 and 18 months old, respectively. Parents were considered to have agreed to participate in the study if they returned the questionnaire to the Ministry of Health, Labor and Welfare. The response rates of the first survey (at 6 months) were 87.8 and 88.1% for the first and second cohorts, respectively; for the second survey (at 18 months), they were 82.0 and 76.2%, respectively.

**Figure 1:**
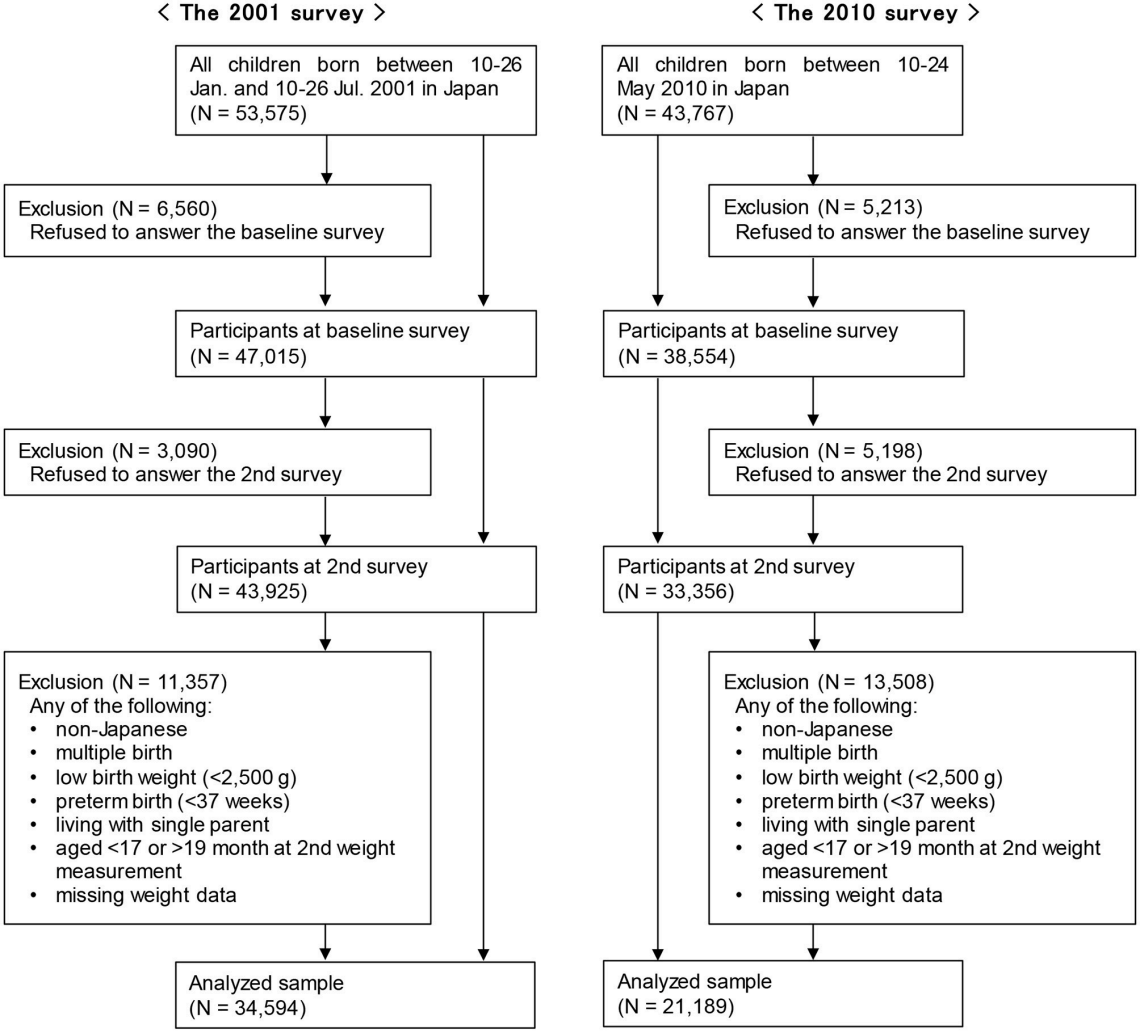
Study population.

We restricted our analyses to 43,925 and 33,356 respondents of the first and second cohorts, respectively, who completed both surveys (Figure 1). We excluded those who met the following criteria: non-Japanese parents; multiple births; low birth weight (<2,500 g); preterm birth (delivery at <37 gestational weeks); living with a single parent; aged <17 or >19 months at the 2nd weight measurement; or missing weight data. A total of 34,594 and 21,189 respondents of the first and second cohorts, respectively, were included in the analysis. Infants living with single parents were excluded to separate the effect of SES and single parent households on weight faltering as the SES of single-parent households is low (16).

We obtained permission from the Ministry of Health, Labor and Welfare to use the survey data (approval no. 0301-2). The study protocol was approved by the Ethics Committee of the National Center for Child Health and Development (no. 1533).

### Outcome: weight faltering

Data on birth weight were extracted from the vital statistics birth record lists. Data on weight at 18 months were obtained from the second survey, in which the parents provided the infants' weight measurements (to the nearest 0.1 kg) and measurement dates according to the 18-month health checkup that all Japanese families must undergo. Using the 2006 World Health Organization's (WHO) Child Growth Standards, the weights were converted to Z-scores (19). Conditional weight gain was calculated using the thrive index method (3), where the thrive index is the change in weight Z-scores from birth to 18 months, adjusted for regression to the mean. The thrive index compares an infant's current weight with the predicted weight, based on their previous weight. Positive and negative thrive indices denote weight gains faster and slower than the average, respectively. Cases of weight faltering were defined as infants whose thrive index was below the 5th percentile (3).

### Predictor: parental socioeconomic status

Data on household income and parental education were obtained from the first and second surveys, respectively. Equalized household income was calculated by dividing the household income in the past year by the square root of the number of persons living in the household and was categorized as quartiles. Educational level was subdivided into junior high school, high school, some college, and college or greater.

### Covariates

Infant sex as well as the mother's and father's ages was obtained from birth records; the number of siblings was derived from the first survey. Breastfeeding exclusiveness, worries about child rearing, the use of childcare services, the father's involvement in childcare (all assessed in the first survey), and illness(es) in the infant (assessed in the second survey) were also used as covariates. Breastfeeding exclusiveness was categorized into exclusive breastfeeding (only breast milk), mixed feeding (both, breast and formula milk), and exclusive formula-feeding (only formula milk) (20). Illness(es) in the infant, which could be associated with weight faltering, were identified when the parents reported visiting a physician in the past year for allergic diseases (asthma, food allergy, or atopic dermatitis) or congenital diseases. We calculated scores for the father's involvement in childcare and categorized them as low, middle, and high (21).

### Statistical analyses

We analyzed the two cohorts separately. Baseline characteristics are described as n (%), and the incidence of weight faltering by SES was calculated. Logistic regression analysis was used to estimate the odds ratios (ORs) and their 95% confidence intervals (CIs) of weight faltering by levels of SES indicators. Models were constructed separately for each SES indicator, based on the known correlations between household income and parental education (22) and adjusted for covariates. Variables with missing data were dummy-coded using the missing-indicator method (23). We examined whether socioeconomic inequalities in weight faltering changed between 2001 and 2010 by comparing the relative index of inequality (RII), which is frequently used as an inequality index in epidemiological studies (24). The RII represents the ratio of estimated values of infant weight faltering for the poorest or least educated parent compared with the richest or most educated parent in the population, while taking into consideration all the other income or education subgroups—using a regression model (24). Finally, we conducted three sensitivity analyses to evaluate the robustness of the results: a multiple imputation method for missing data (Supplemental Methods) (25, 26), different cut-off values for the thrive index to define cases of weight faltering below the 2.5th percentile or 10th percentile, and treating income as a continuous variable (Income was logarithmically transformed due to a skewed distribution).

All statistical tests were two-sided, with a 5% significance level. Most analyses were conducted using SAS version 9.3 for Windows (SAS Inc., Cary, NC, USA); the RII was calculated using HD^*^ Calc version 1.2.4 (National Cancer Institute, Bethesda, MD, US).

## Results

### Baseline characteristics

Sex, the number of siblings, and income quartiles were not substantially different between the two cohorts (Table 1). About half of infants did not have siblings. The mother's and father's ages as well as their educational level were higher for infants of the second cohort when compared to those of the first cohort. This reflected a high rate of attaining higher education and the corresponding increase in the prevalence of childbearing at older ages (16). The proportions of infants who were mixed-fed or had illnesses were lower in the second cohort. Higher educated parents had higher median income compared with lower educated parents (Supplemental Table 1).

**Table 1:** Characteristics of the study population by survey year.

**Characteristics**	**2001 (*****N*** = **34,594)**		**2010 (*****N*** = **21,189)**	
	***n***	**%**	***n***	**%**
**INFANT'S SEX**				
Male	18,063	52.2	11,031	52.1
Female	16,531	47.8	10,158	47.9
**NO. OF SIBLINGS**				
0	17,192	49.7	10,349	48.8
1	12,833	37.1	8,009	37.8
2	3,884	11.2	2,403	11.3
≥3	685	2.0	428	2.0
**MOTHER'S AGE (YEARS)**				
≤24	3,208	9.3	1,190	5.6
25–29	12,101	35.0	5,374	25.4
30–34	13,900	40.2	8,343	39.4
35–39	4,705	13.6	5,307	25.1
≥40	680	2.0	975	4.6
**FATHER'S AGE (YEARS)**				
≤24	2,083	6.0	760	3.6
25–29	8,915	25.8	4,055	19.1
30–34	12,994	37.6	7,452	35.2
35–39	7,415	21.4	6,126	28.9
≥40	3,187	9.2	2,796	13.2
**INCOME QUARTILE^a^**				
1st (lowest)	7,213	20.9	4,276	20.2
2nd	8,137	23.5	5,032	23.8
3rd	8,541	24.7	5,206	24.6
4th (highest)	8,679	25.1	5,454	25.7
Missing	2,024	5.9	1,221	5.8
**MOTHER'S EDUCATION**				
Junior high school	1,604	4.6	800	3.8
High school	13,400	38.7	5,535	26.1
Some college	14,594	42.2	8,844	41.7
College or greater	4,905	14.2	5,932	28.0
Others or missing	91	0.3	78	0.4
**FATHER'S EDUCATION**				
Junior high school	2,602	7.5	1,219	5.8
High school	13,475	39.0	6,293	29.7
Some college	5,469	15.8	3,864	18.2
College or greater	12,758	36.9	9,582	45.2
Others or missing	290	0.8	231	1.1
**BREASTFEEDING EXCLUSIVENESS**				
Exclusive formula feeding	1,815	5.3	550	2.6
Exclusive breastfeeding	7,861	22.7	7,636	36.0
Mixed feeding	24,676	71.3	12,872	60.8
Missing	242	0.7	131	0.6
**INFANT'S ILLNESS (VISIT TO DOCTORS)^b^**				
No	25,758	74.5	17,895	84.5
Yes	7,990	23.1	2,608	12.3
Missing	846	2.5	686	3.2
**WORRIES ABOUT CHILD REARING**				
Few	12,998	37.6	8,189	38.7
Many or a few	21,521	62.2	12,976	61.2
Missing	75	0.2	24	0.1
**THE USE OF CHILDCARE SERVICES**				
No	33,262	96.2	20,354	96.1
Yes	1,327	3.8	824	3.9
Missing	5	0.0	11	0.1
**FATHER'S INVOLVEMENT IN CHILDCARE**				
Low	5,133	14.8	3,279	15.5
Middle	22,363	64.6	13,871	65.5
High	5,791	16.7	3,480	16.4
Missing	1,307	3.8	559	2.6

a *Annual household income divided by the square root of the number of household members*.

b *Illness including allergic diseases (asthma, food allergy, and atopic dermatitis) and congenital diseases*.

### Weight

The mean birth weights were 3,121.1 g (standard deviation [SD], 338.1 g) and 3,096.0 g (SD, 330.0 g) in the first and second cohorts, respectively. The mean weight Z-scores at birth were −0.39 (SD, 0.72) and −0.44 (SD, 0.74) for the first and second cohorts, respectively, indicating that Japanese infants were fairly smaller than the WHO standard population at birth. The mean weights at 18 months were 10.6 kg (SD, 1.1 kg) and 10.4 kg (SD, 1.1 kg) in the first cohort and second cohorts, respectively, and the mean weight Z-scores at 18 months were −0.03 (SD, 0.87) and −0.14 (SD, 0.84), respectively, indicating that our sample was slightly smaller than the WHO standard population at 18 months.

Table 2 shows the weight Z-scores at birth/18 months, thrive indices, and the cases of weight faltering by SES indicators for both cohorts. The rate ratios of weight faltering for infants in the lowest- vs. highest-income households were 1.4 and 1.5 for the first and second cohorts, respectively. No clear gradient was observed for parental education in both cohorts.

**Table 2:** Weight z scores at birth and 18 months, thrive index values, and cases of weight faltering according to parental socioeconomic status by survey year.

	**Weight z score^a^ at birth Mean (SD)**				**Weight z score^a^ at 18 months Mean (SD)**				**Thrive index^b^ Mean (SD)**				**Weight faltering^c^ % of cases**	
**Parental socioeconomic status**	**2001**		**2010**		**2001**		**2010**		**2001**		**2010**		**2001**	**2010**
**INCOME QUARTILE**														
1st (lowest)	−0.35	(0.74)	−0.41	(0.74)	−0.03	(0.90)	−0.16	(0.88)	−0.01	(0.85)	−0.03	(0.82)	5.7	6.1
2nd	−0.37	(0.73)	−0.43	(0.70)	−0.06	(0.88)	−0.14	(0.85)	−0.04	(0.84)	−0.01	(0.80)	5.7	5.7
3rd	−0.40	(0.72)	−0.45	(0.71)	−0.03	(0.85)	−0.14	(0.83)	0.002	(0.80)	0.01	(0.78)	4.5	4.3
4th (highest)	−0.43	(0.71)	−0.47	(0.70)	0.01	(0.85)	−0.11	(0.82)	0.05	(0.81)	0.04	(0.76)	4.1	4.0
Rate ratio (lowest/highest)													1.4	1.5
**MOTHER'S EDUCATION**														
Junior high school	−0.38	(0.74)	−0.41	(0.72)	0.02	(0.89)	−0.10	(0.90)	0.04	(0.86)	0.02	(0.84)	5.2	5.8
High school	−0.38	(0.74)	−0.44	(0.72)	−0.04	(0.89)	−0.12	(0.85)	−0.01	(0.84)	0.01	(0.80)	5.2	5.0
Some college	−0.39	(0.72)	−0.44	(0.72)	−0.04	(0.86)	−0.15	(0.84)	−0.01	(0.82)	−0.01	(0.79)	5.0	5.2
College or greater	−0.41	(0.71)	−0.46	(0.69)	0.01	(0.86)	−0.14	(0.82)	0.04	(0.81)	0.005	(0.77)	4.4	4.6
Rate ratio (lowest/highest)													1.2	1.2
**FATHER'S EDUCATION**														
Junior high school	−0.41	(0.75)	−0.41	(0.73)	−0.02	(0.90)	−0.10	(0.93)	0.02	(0.85)	0.06	(0.82)	5.1	5.1
High school	−0.39	(0.72)	−0.44	(0.73)	−0.03	(0.89)	−0.11	(0.87)	0.003	(0.84)	0.004	(0.81)	5.2	5.4
Some college	−0.39	(0.74)	−0.43	(0.71)	−0.06	(0.87)	−0.15	(0.85)	−0.03	(0.83)	−0.02	(0.78)	5.1	5.1
College or greater	−0.38	(0.72)	−0.45	(0.70)	−0.02	(0.85)	−0.14	(0.83)	0.004	(0.81)	−0.004	(0.77)	4.8	4.7
Rate ratio (lowest/highest)													1.1	1.1

*SD, standard deviation*.

a *Weight z score is the number of standard deviations each infant's weight is away from the median weight for the same age and sex in WHO standard population*.

b *A thrive index is the change in weight z scores from birth to 18 months, adjusted for the infant's initial weight*.

c *Cases of weight faltering were defined as infants whose thrive index was below the 5th percentile*.

### Socioeconomic status and the risk of weight faltering

In the logistic regression analysis (Table 3), infants from low-income families (1st and 2nd quartiles) were at higher risk of weight faltering than those from higher-income families (4th quartile) in the first cohort. The findings remained significant after adjustments for covariates (OR 1.29, 95% CI [1.10, 1.52]; OR 1.29, 95% CI [1.11, 1.50]; and OR 1.03, 95% CI [0.89, 1.20] for the for 1st, 2nd, and 3rd quartiles, respectively); we observed a dose-response association (*p* for trend < 0.001). Similar associations were observed in the second cohort; the findings remained significant after adjustments for covariates (OR 1.27, 95% CI [1.03, 1.56]; OR 1.24, 95% CI [1.02, 1.50]; and OR 1.00, 95% CI [0.82, 1.21] for 1st, 2nd, and 3rd quartiles, respectively; *p* for trend = 0.006). No significant associations were observed between parental education and weight faltering in the crude model for both cohorts. However, after adjustments for covariates, a significant association between lower parental education and weight faltering appeared for the first but not the second cohort (high school, mother: OR 1.18, 95% CI [1.01, 1.39]; high school, father: OR 1.13, 95% CI [1.01, 1.27]).

**Table 3:** Odds ratios (ORs) and inequality indices for weight faltering according to parental socioeconomic status by survey year.

	**2001**				**2010**			
	**Crude model**		**Adjusted model^a^**		**Crude model**		**Adjusted model^a^**	
**Parental socioeconomic status**	**OR**	**(95% CI)**	**OR**	**(95% CI)**	**OR**	**(95% CI)**	**OR**	**(95% CI)**
**INCOME QUARTILE**								
1st (lowest)	1.42	(1.23, 1.64)^*^	1.29	(1.10, 1.52)^*^	1.55	(1.29, 1.86)^*^	1.27	(1.03, 1.56)^*^
2nd	1.41	(1.22, 1.62)^*^	1.29	(1.11, 1.50)^*^	1.44	(1.21, 1.73)^*^	1.24	(1.02, 1.50)^*^
3rd	1.09	(0.94, 1.27)	1.03	(0.89, 1.20)	1.08	(0.89, 1.31)	1.00	(0.82, 1.21)
4th (highest)	1.00		1.00		1.00		1.00	
*p* for trend	<0.001		<0.001		<0.001		0.006	
RII	1.66 (1.36, 1.96)^*^				1.86 (1.42, 2.31)^*^			
**MOTHER'S EDUCATION**								
Junior high school	1.19	(0.92, 1.54)	1.25	(0.95, 1.63)	1.26	(0.91, 1.74)	1.12	(0.80, 1.57)
High school	1.17	(0.99, 1.37)	1.18	(1.01, 1.39)^*^	1.10	(0.92, 1.30)	1.04	(0.87, 1.24)
Some college	1.14	(0.98, 1.33)	1.14	(0.98, 1.33)	1.13	(0.97, 1.32)	1.09	(0.93, 1.27)
College or greater	1.00		1.00		1.00		1.00	
*p* for trend	0.051		0.026		0.147		0.521	
RII	0.96 (0.76, 1.16)				1.15 (0.94, 1.37)			
**FATHER'S EDUCATION**								
Junior high school	1.07	(0.88, 1.30)	1.12	(0.91, 1.36)	1.08	(0.82, 1.42)	1.01	(0.77, 1.34)
High school	1.10	(0.99, 1.23)	1.13	(1.01, 1.27)^*^	1.16	(1.00, 1.34)^*^	1.11	(0.96, 1.29)
Some college	1.06	(0.92, 1.23)	1.10	(0.95, 1.28)	1.07	(0.91, 1.28)	1.07	(0.90, 1.27)
College or greater	1.00		1.00		1.00		1.00	
*p* for trend	0.100		0.042		0.040		0.165	
RII	1.00 (0.73, 1.27)				1.09 (0.92, 1.25)			

*CI, confidence interval; RII, relative index of inequality*.

a *Adjusted for covariates including infant's sex, no. of siblings, mother's age, and father's age, breastfeeding exclusiveness, infant's illness, worries about child rearing, the use of childcare services, and father's involvement in childcare*.

* *P < 0.05*.

The RII for income was 1.66 (95% CI [1.36, 1.96] in 2001 and 1.86 (95% CI [1.42, 2.31]) in 2010, indicating that the magnitude of income-related inequalities in weight faltering did not change (*p* = 0.545).

### Sensitivity analysis

The sensitivity analyses confirmed the robustness of the results. We found similar results when using a multiple-imputation method for handling missing data (Supplemental Table 2) and when using different cut-off values for the thrive index to define cases of weight faltering (thrive index below the 2.5th centile or 10th percentile) (Supplemental Table 3). We also found similar associations when treating income as a continuous variable; weight faltering was negatively associated with income both in the 2001 (OR per ten-thousand yen 0.90, 95% CI [0.82, 0.99]) and 2010 cohorts (OR per ten-thousand yen 0.80, 95% CI [0.70, 0.91]).

## Discussion

Using two nationally representative samples of Japanese infants and their families, we found that infants from lower-income families, but not parental education, had a greater risk of weight faltering in both cohorts and that income-related inequalities in weight faltering did not change from 2001 to 2010.

Our results suggest that household income affects weight faltering more so than parental education. Because parental education is positively correlated to household income (22), factors that are exclusively related to household income are likely associated with the risk of weight faltering. Possible candidates for the association between household income and weight faltering include the inadequate provision of food due to financial reasons or neglect due to unmeasured longer working hours to compensate for low income, which can lead to undernutrition (27).

Poor access to adequate amounts and types of food may mediate the association between household income and weight faltering in Japan. According to the Ministry of Health, Labor and Welfare, roughly 1 in 6 Japanese children (16.3%) <18 years old lived in relative poverty (defined as households with income below half of the national median) in 2012 (28). One Japanese community-based study reported (29) that the daily food expenses of a household in relative poverty were on average, 340 Japanese Yen (approximately 3 US dollars) per person, which is about 40% of the total household income (about 850 Japanese Yen) (30). Moreover, about 80% of households in relative poverty often or sometimes refrained from purchasing foods due to economic reasons (29). According to the 2015 National Nutrition Survey on Preschool Children (31), parents with subjective low economic status reported lower frequencies of fish, soybean, vegetable, and fruit consumption for their children when compared to their higher-status counterparts. Because healthy foods are relatively more expensive than processed foods, parents with low subjective economic status might not be able to afford them (29).

Neglect can also mediate the association between household income and weight faltering as it is known to have similar psychosocial risk factors (27). Previous studies suggested that poverty contributes to neglect (32). Neglectful parents tend to abandon the responsibility of feeding their infants and ignore their hunger signals and other emotional/physical needs (33). Physical neglect leads to weight faltering through undernutrition related to feeding problems (33). The estimated worldwide prevalence of neglect ranges widely, from 1.4 to 80.1%, as neglect is difficult to measure in children (34). In Japan, only one national survey on abuse cases reported an estimated prevalence of maltreatment of 1.83 per 1,000 children aged 2 weeks to 11 months in 2000 (35); of all maltreated children, 81% were neglected and 88% were abused by their mothers who were the primary caregivers. The actual prevalence of neglect in Japan is considered to be higher as child abuse cases are underreported (36).

We also show that the income-related inequalities in weight faltering persisted from 2001 to 2010. In Japan, only few anti-poverty programs, especially those targeting children, have been implemented as Japan has long been considered an egalitarian society (37). Although it has been increasingly acknowledged that considerable income inequality exists in Japan (28), public support for children remains limited and considerably lower than that for the elderly; for example, pension and medical insurance for the elderly accounted for about 80% of Japan's social security benefits in 2016, while child-related assistance in the same year accounted for only about 5% (17). Moreover, the proportion of family-related social expenditures of the gross domestic product in Japan has been lower than that of other developed countries (17). Thus, the persistent lack of social security for families with children could have resulted in the maintained income-related inequalities in weight faltering. We observed significant association between low parental education and weight faltering in 2001 only. Moreover, the parental educational level was higher in the second than in the first cohort. The increasing rate of attaining higher education might have reduced the effect of parental education on weight faltering.

This study's strengths include its large nationally representative sample, a high response rate, and the comparison of data collected in different years. To the best of our knowledge, we are the first to show income disparities in weight faltering among Japanese infants. However, the study also has limitations. First, infants who are at risk for weight faltering might be overrepresented in lower-income groups due to the known association between low SES and worse birth outcomes (38). However, we excluded infants with low birth weights and preterm births. Second, we could not identify when weight faltering occurred due to the long survey interval (birth to 18 months). We also could not exclude cases of acute weight loss due to gastrointestinal complaints (diarrhea, vomiting, or dehydration) and communicable diseases in the second survey due to incomplete data reporting. Finally, we lacked data on food access and neglect that might have helped us assess the mediating mechanisms between income inequality and weight faltering.

These results have several implications. Expanding expenditures on family-related policies may be effective in reducing income inequality and thereby inequalities in weight faltering. Food support programs for low-income families, especially for those with infants, would help them gain access to adequate amounts and types of food. In some developed countries (e.g., the USA), government programs provide food, nutrition counseling, and healthcare screening. These programs can protect infants from low-income families against negative poverty-related health and growth consequences (39). Although non-profit sector food support programs have been spreading across Japan (29), policy makers should consider implementing such programs in the public sector.

In conclusion, in contrast to previous studies in UK and Denmark, infants from lower-income families had a greater risk of weight faltering in Japan than those from higher-income families. Income-related inequalities in weight faltering did not change over time. Further prospective studies should evaluate the underlying mechanisms by which income inequality adversely affects weight faltering in Japan.

## Author contributions

YK and TF conceptualized and designed the study. YK analyzed data and wrote first draft. YK, TF, YY, and TK interpreted results and revised manuscript. All authors approved the final manuscript as submitted and agree to be accountable for all aspects of the work.

### Conflict of interest statement

The authors declare that the research was conducted in the absence of any commercial or financial relationships that could be construed as a potential conflict of interest.
