# Supplementary material for: Parental Socioeconomic Status and Weight Faltering in Infants in Japan
Source: Front Pediatr. 2018 May 1;6:127. doi: 10.3389/fped.2018.00127 (PMC5938368; doi:10.3389/fped.2018.00127)
Supplement: Supplementary file 1 [file Presentation_1.PDF]

Supplemental Methods: details of a multiple imputation method

The percentage of missing data in each cohort was less than 6% for all variables discussed in this article. In total, 4331 records (12.5 %) for the first cohort and 2584 records (12.2%) for the second cohort were incomplete. The imputation was performed using the fully conditional specification in SAS PROC MI.[25] The results across 200 imputed data sets were combined using Rubin's rules.[26]

25. van Buuren S. Multiple imputation of discrete and continuous data by fully conditional specification. Stat Methods Med Res. 2007; 16:219-242.

26. Rubin DB. Multiple imputation for nonresponse in surveys. New York: John Wiley & Sons, Inc.; 1987.

Supplemental Table 1. Association between parental education and household income.

Parental education	Household income, ten-thousand yen median [interquartile range]			
	2001		2010	
Mother's education				
Junior high school	216	[150-292]	230	[165-305]
High school	269	[198-358]	269	[200-359]
Some colledge	333	[242-450]	325	[241-445]
Colledge or greater	416	[301-566]	424	[300-580]
Father's education				
Junior high school	219	[156-297]	231	[171-317]
High school	277	[202-375]	279	[205-381]
Some colledge	289	[212-390]	298	[226-412]
Colledge or greater	382	[289-516]	396	[289-548]

Supplemental Table 2. A comparison of odds ratios (ORs) of parental socioeconomic status on weight faltering using missing-indicator and multiple-imputation methods.

	2001				2010			
	Missing indicator method		Multiple imputation method		Missing indicator method		Multiple imputation method	
Parental socioeconomic status	OR	(95% CI)[a]	OR	(95% CI)[a]	OR	(95% CI)[a]	OR	(95% CI)[a]
Income quartile								
1st (lowest)	1.29	(1.10, 1.52)*	1.28	(1.09, 1.51)*	1.27	(1.03, 1.56)*	1.26	(1.02, 1.55)*
2nd	1.29	(1.11, 1.50)*	1.28	(1.10, 1.50)*	1.24	(1.02, 1.50)*	1.23	(1.02, 1.50)*
3rd	1.03	(0.89, 1.20)	1.03	(0.89, 1.20)	1.00	(0.82, 1.21)	1.00	(0.82, 1.21)
4th (highest)	1.00		1.00		1.00		1.00	
Mother's education								
Junior high school	1.25	(0.95, 1.63)	1.25	(0.96, 1.63)	1.12	(0.80, 1.57)	1.12	(0.80, 1.57)
High school	1.18	(1.01, 1.39)*	1.18	(1.01, 1.39)*	1.04	(0.87, 1.24)	1.04	(0.87, 1.24)
Some colledge	1.14	(0.98, 1.33)	1.14	(0.98, 1.33)	1.09	(0.93, 1.27)	1.09	(0.93, 1.27)
Colledge or greater	1.00		1.00		1.00		1.00	
Father's education								
Junior high school	1.12	(0.91, 1.36)	1.11	(0.91, 1.36)	1.01	(0.77, 1.34)	1.02	(0.77, 1.35)
High school	1.13	(1.01, 1.27)*	1.13	(1.01, 1.27)*	1.11	(0.96, 1.29)	1.11	(0.96, 1.29)
Some colledge	1.10	(0.95, 1.28)	1.10	(0.95, 1.27)	1.07	(0.90, 1.27)	1.07	(0.90, 1.27)
Colledge or greater	1.00		1.00		1.00		1.00	

CI = confidence interval; RII = relative index of inequality.

[a]Adjusted for covariates including infant's sex, no. of siblings, equivalized, mother's age, and father's age, breastfeeding exclusiveness, infant's illness, worries about child rearing, the use of childcare services, and father's involvement in childcare.

*$P < 0.05$.

Supplemental Table 3. A comparison of odds ratios (ORs) of parental socioeconomic status on weight faltering using different cut-off values.

	2001				2010			
	TI < 2.5 percentile		TI < 10 percentile		TI < 2.5 percentile		TI < 10 percentile	
Parental socioeconomic status	case, %	OR (95% CI)[a]	case, %	OR (95% CI)[a]	case, %	OR (95% CI)[a]	case, %	OR (95% CI)[a]
Income quartile								
1st (lowest)	2.7	1.13 (0.89, 1.42)	10.9	1.21 (1.08, 1.37)*	3.3	1.54 (1.15, 1.85)*	11.4	1.22 (1.05, 1.42)*
2nd	2.8	1.22 (0.99, 1.50)	11.3	1.28 (1.15, 1.43)*	2.8	1.36 (1.03, 1.78)*	11.0	1.20 (1.05, 1.38)*
3rd	2.3	1.04 (1.04, 1.29)	9.4	1.07 (0.96, 1.19)	2.1	1.04 (0.79, 1.37)	9.2	1.03 (0.90, 1.18)
4th (highest)	2.0	1.00	8.4	1.00	1.9	1.00	8.5	1.00
Mother's education								
Junior high school	2.5	1.30 (0.88, 1.90)	9.7	1.12 (0.92, 1.36)	3.3	1.21 (0.77, 1.88)	11.4	1.17 (0.91, 1.49)
High school	2.8	1.41 (1.12, 1.78)*	10.7	1.22 (1.09, 1.37)*	2.5	0.99 (0.77, 1.27)	10.3	1.07 (0.94, 1.22)
Some colledge	2.4	1.21 (0.96, 1.52)	9.8	1.11 (0.99, 1.24)	2.6	1.08 (0.87, 1.34)	10.0	1.04 (0.93, 1.17)
Colledge or greater	2.0	1.00	8.9	1.00	2.3	1.00	9.5	1.00
Father's education								
Junior high school	2.2	0.95 (0.71, 1.28)	10.3	1.14 (0.99, 1.32)	2.9	1.16 (0.80, 1.68)	9.8	0.99 (0.81, 1.22)
High school	2.7	1.16 (0.99, 1.36)	10.4	1.14 (1.05, 1.24)*	2.8	1.14 (0.93, 1.40)	10.7	1.10 (0.98, 1.22)
Some colledge	2.7	1.18 (0.96, 1.45)	10.5	1.16 (1.04, 1.29)*	2.4	1.04 (0.81, 1.33)	10.0	1.04 (0.91, 1.18)
Colledge or greater	2.3	1.00	9.4	1.00	2.3	1.00	9.7	1.00

CI = confidence interval; RII = relative index of inequality.

[a]Adjusted for covariates including infant's sex, no. of siblings, equivalized, mother's age, and father's age, breastfeeding exclusiveness, infant's illness, worries about child rearing, the use of childcare services, and father's involvement in childcare.

*$P < 0.05$.
